# Development and Validation of a Robust RP‐HPLC Method for Quantifying Dasatinib in Self‐Microemulsifying Drug Delivery Systems

**DOI:** 10.1155/ianc/4376722

**Published:** 2026-02-01

**Authors:** Nitin V. Kokare, Rohit R. Shah, Rana Salman Saad Al-Rashidi, Kuntal Das, Syed Mohammed Basheeruddin Asdaq, Walaa F. Alsanie, Abdulhakeem S. Alamri, Majid Alhomrani, Amal F. Alshammary, Syed Arif Hussain, Syed Imam Rabbani, Hanumantharayappa Bylappa, Sultan Alshehri

**Affiliations:** ^1^ Department of Quality Assurance, Appasaheb Birnale College of Pharmacy, Sangli, 416416, Maharashtra, India; ^2^ Department of Pharmaceutics, Appasaheb Birnale College of Pharmacy, Sangli, 416416, Maharashtra, India; ^3^ Department of Pharmacy, King Khalid University Hospital, Riyadh, 11472, Saudi Arabia, ksu.edu.sa; ^4^ Sree Siddaganga College of Pharmacy, 3rd Block Mahalakshmi Nagar Near Railway Gate 80 feet Road Batawadi, Tumkur, 572013, Karnataka, India; ^5^ Department of Pharmacy Practice, College of Pharmacy, AlMaarefa University, Ad Diriyah, 13713, Saudi Arabia, um.edu.sa; ^6^ Department of Clinical Laboratory Sciences, The Faculty of Applied Medical Sciences, Taif University, Taif, 21944, Saudi Arabia, tu.edu.sa; ^7^ Department of Clinical Laboratory Sciences, College of Applied Medical Sciences, King Saud University, Riyadh, 11472, Saudi Arabia, ksu.edu.sa; ^8^ Department of Respiratory Care, College of Applied Sciences, AlMaarefa University, Dariyah, Riyadh, 13713, Saudi Arabia, um.edu.sa; ^9^ Department of Pharmacology and Toxicology, College of Pharmacy, Qassim University, Buraydah, 51452, Saudi Arabia, qu.edu.sa; ^10^ Department of Pharmacology, Mallige College of Pharmacy, #71, Silvepura, Chikkabanavara Post, Bangalore, 560090, India, mallige.ac.in; ^11^ Department of Pharmaceutics, College of Pharmacy, King Saud University, Riyadh, 11451, Saudi Arabia, ksu.edu.sa

**Keywords:** dasatinib, drug design, formulation, reverse-phase HPLC, stability, validation

## Abstract

Dasatinib, a therapy for chronic myeloid leukemia, suffers from poor bioavailability. Self‐microemulsifying drug delivery systems (SMEDDSs) are used to improve its dissolution. This study aimed to develop and validate a novel reverse‐phase high‐performance liquid chromatography (RP‐HPLC) method for quantifying dasatinib in SMEDDS formulations. The RP‐HPLC method utilized a mobile phase of methanol and 0.1% trifluoroacetic acid (55:45, v/v) and identified a peak wavelength for dasatinib at 324 nm. SMEDDS formulations comprised Capryol 90, Transcutol HP, and Tween 40. The method was validated according to ICH guidelines, demonstrating excellent linearity (*R* ^2^ = 0.9993), accuracy (recovery between 98% and 101%), and precision (relative standard deviation of 0.73%). It also showed stability and reliability with limits of detection and quantification of 0.17 and 0.50 µg/mL, respectively. This RP‐HPLC method meets all validation criteria and provides a robust, cost‐effective tool for analyzing dasatinib in SMEDDS formulations.

## 1. Introduction

Chronic myeloid leukemia (CML), also referred to as chronic myelogenous leukemia, is a cancer of white blood cells. Dasatinib (also referred to as BMS‐354825) is a powerful dual Src/Abl kinase inhibitor, belonging to the class of thiazole‐based drugs. It has been approved for the treatment of CML, primarily serving as an alternative for patients who do not respond to or cannot tolerate imatinib (commonly used as first‐line therapy in CML) [1]. Tyrosine kinase inhibitors (TKIs) like dasatinib work by blocking specific intracellular signaling pathways that are involved in receptor‐mediated growth signaling, thereby inhibiting the proliferation of malignant cells [2, 3]. Dasatinib is administered orally in tablet or capsule form, but its formulations face challenges related to poor bioavailability (14%–34%), which can limit therapeutic efficacy [4].

To overcome the issue of low solubility and bioavailability, self‐microemulsifying drug delivery systems (SMEDDSs) have emerged as an effective pharmaceutical solution [5]. SMEDDSs are prepared by dissolving the drug in oil and then adding surfactants and cosurfactants. These systems form stable, isotropic mixtures that are clear and transparent in appearance [6]. Due to their emulsifying properties, SMEDDSs facilitate targeted drug delivery to specific sites within the body [7]. Furthermore, SMEDDS offers improved dosage control, contributing to better patient compliance. Other benefits of SMEDDSs include enhanced intestinal solubility, increased drug permeability, prolonged gastric resistance, and flexibility in excipient selection [6, 8].

**FIGURE 1:**
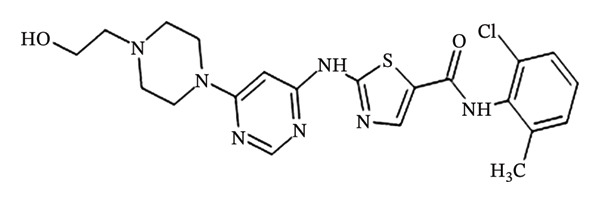
Chemical structure of dasatinib [1, 9].

Dasatinib’s chemical name is N‐(2‐chloro‐6‐methylphenyl), and its molecular formula is C_22_H_26_ClN_7_O_2_S, with a molecular weight of 488.0055. The structural formula is represented as N‐(2‐chloro‐6‐methylphenyl)‐2‐(6‐(4‐(2‐hydroxyethyl)piperazin‐1‐yl)‐2‐methylpyrimidin‐4‐yl)amino)thiazole‐5‐carboxamide monohydrate [9]. A structural depiction of dasatinib is shown in Figure 1.

The development of a new analytical method typically involves several stages, including the adaptation of existing techniques, tailoring specific adjustments for novel applications, and the creation of a high‐performance liquid chromatography (HPLC) method for drug identification [10, 11]. Method validation, as outlined by Yashin and Yashin, is an essential procedure for assessing the suitability of an analytical technique in identifying drugs within pharmaceutical formulations [10].

Previous studies suggest that the quantification of dasatinib is most effectively achieved through chromatographic and mass spectrometric techniques across various pharmaceutical formulations, such as injectables, tablets, and pure drug samples [1, 12]. Dasatinib has been successfully identified in tablet and injectable forms using reverse‐phase HPLC (RP‐HPLC) methods [13]. Validated results indicate that this method is reliable, allowing for routine analysis of dasatinib in diverse pharmaceutical formulations [13, 14].

Research has also demonstrated dasatinib’s stability under various stress conditions, such as acidic, basic, oxidative, and hydrolytic environments, using reverse‐phase liquid chromatographic methods [15]. Gradient HPLC analysis has been employed for the determination of dasatinib and its impurities, proving more effective for these assessments [15, 16]. However, these methods have been criticized for being time‐consuming (often requiring hours), costly, and complex, with multiple steps necessary for validating the active pharmaceutical ingredient [14].

Although dasatinib can be detected in biological samples using chromatographic techniques like HPLC or LC–MS [13, 14], there is a gap in the literature regarding analytical methods specifically tailored for dasatinib in SMEDDSs (Table 1). Furthermore, such methods should apply to both preserved and freshly prepared samples, to facilitate method development for investigational purposes [18, 19].

**TABLE 1:** Comparative evaluation of published and proposed analytical methods.

The published method shows the following disadvantages	The proposed method offers the following advantages
Runtime 6.4 [17]	Runtime 4.97, the proposed method gives decreased runtime
LOD 2.83 µg/mL [17]	LOD 0.17 µg/mL, lowered LOD
LOQ 9.41 µg/mL [17]	LOQ 0.50 µg/mL, lowered LOQ
The current method is not only time‐intensive but also fails to deliver results with the desired precision and accuracy Published methods do not address drug analysis from SMEDDS	The streamlined method ensures high precision and accuracy in the results The proposed method is specifically developed to analyze the drug from SMEDDS

To address this critical analytical gap, the present study introduces and validates a novel, rapid, and stability‐indicating RP‐HPLC method specifically designed for quantifying dasatinib in complex lipid‐based SMEDDS matrices. Unlike previous studies that focused on tablet or bulk drug analysis, our work emphasizes method optimization for excipient‐rich nanoemulsion systems, which often cause chromatographic interferences. The developed method employs a simplified mobile phase without buffer, achieves a shorter retention time (RT) (∼4.9 min), and demonstrates high recovery (≥ 99%) and precision (%RSD < 2%). Moreover, this approach enables reliable quantification of dasatinib during formulation screening and stability assessment, making it distinct from previously reported techniques.

A comparative overview (Table 1) of multiple published HPLC methods with the proposed one highlights its analytical efficiency, cost‐effectiveness, and practical applicability for lipid‐based formulations. Thus, this work not only bridges an unaddressed analytical challenge but also provides a validated, formulation‐specific tool for dasatinib quantification in SMEDDSs.

Therefore, the current study aims to validate a novel analytical technique for dasatinib quantification in SMEDDSs. This approach employs diode array detection (DAD) coupled with HPLC, providing a rapid, user‐friendly, and practical solution for routine pharmaceutical investigations.

## 2. Materials and Methods

### 2.1. Chemicals and Reagents

Dasatinib powder (99.45% w/w) was obtained from Shilpa Medicare Ltd., Raichur, Karnataka, India. HPLC‐grade methanol and trifluoroacetic acid (TFA) were purchased from Merck Specialties Pvt. Ltd., Mumbai, India. Double‐distilled water was produced using the Milli‐Q Water System. Before using, the mobile phase was degassed by ultrasonication for 20 min and filtered through a 0.45‐µm, 47‐mm filter, sourced from Pall India Pvt. Ltd., Mumbai, India. All weighing tasks were carried out using a digital electronic balance (Model ATX‐124) manufactured by Shimadzu, India.

### 2.2. Chromatographic Conditions

Chromatographic analysis was conducted using an Agilent 1260 system, comprising an Agilent diode array detector (model G7115A), auto‐injector (model G7129A), quaternary pump, and HPLC pump degasser (G7111A). Data acquisition and analysis were managed using a chromatography data system. The chromatographic separation was performed using a C18 column having dimensions 4.6 × 150 mm, 5 µm, and 400 bars from Agilent Technologies, OpenLab EZChrom system, with methanol and 0.1% TFA mixed in a 55:45 ratio as the mobile phase. UV detection was conducted at 324 nm, with a flow rate of 1 mL/min. The column was maintained at 30°C, and an injection volume of 20 µL was used. The mobile phase was filtered using a 0.45‐µm Nylon‐66 filter from Pall India Pvt. Ltd., Mumbai, India [20].

### 2.3. Standard Stock Solution Preparation

For dilutions, 5 mg of dasatinib was dissolved in methanol in a 10‐mL volumetric flask and sonicated for 15 min to prepare a standard stock solution. This solution was adjusted to a final concentration of 500 µg/mL.

### 2.4. Preparation of Working Standard

To prepare the working standard, 1.0 mL of the stock solution was transferred to a 10‐mL volumetric flask and diluted with a suitable diluent. The flask was vortexed for one minute to ensure thorough mixing. The final concentration of the working standard was 50 µg/mL.

### 2.5. Validation Procedure

The chromatographic method was validated according to the International Conference on Harmonization’s Q2 (R1) guidelines. Key parameters, including range, precision, detection limit, linearity, accuracy, specificity, and quantification limit, were evaluated during the validation [2].

### 2.6. Linearity

The calibration curve for dasatinib was constructed by diluting the standard stock solution to generate concentrations ranging from 0 to 100 μM. Considering dasatinib’s 99.45% purity, a 500 µg/mL stock solution was prepared. From this stock, aliquots of 0.2, 0.4, 0.6, 0.8, 1.0, and 1.2 mL were transferred to separate 10 volumetric flasks and diluted to final volumes, yielding concentrations of 10, 20, 30, 40, 50, and 60 µg/mL, respectively. These solutions were injected into the HPLC system with consistent injection volumes. The dasatinib peak area ratio was plotted against the nominal concentrations of the standard solutions. The regression line for the standard solutions was calculated using the linear least squares method, confirming linearity. Response factors were also calculated by correlating the peak area to the concentration of each standard. Linearity, accuracy, and precision were further evaluated to determine the range of the method [21, 22].

### 2.7. Precision

Instrumental precision and repeatability were evaluated to assess method accuracy. Six sets of dasatinib reference samples were analyzed to determine the precision of the method. Relative error (RE) and relative standard deviation (%RSD) were calculated to assess precision. The concentrations used for testing were based on the low, mid, and high points of the calibration curve, as recommended by Sadashivaiah et al. [23].

### 2.8. Accuracy/Recovery

Accuracy in the analytical method refers to how closely the test results align with the true value. The method’s accuracy was assessed by analyzing three distinct concentrations of dasatinib. Recovery was calculated based on the observed increase in concentration, and accuracy was expressed as the %RSD of these recoveries [24].

### 2.9. Sensitivity (Limits of Detection [LODs] and Limits of Quantity [LOQs])

The LODs and LOQs are crucial for evaluating the sensitivity of an analytical method. These limits represent the lowest concentrations of analyte that can be consistently detected and quantified with acceptable precision and accuracy [25, 26]. The LOD and LOQ were calculated using the following formulas, where *S* is the slope of the calibration curve and *σ* is the standard deviation of the *y*‐intercept:

(1) LOD=3.3×σS,

(2) LOQ=10×σS.

### 2.10. Robustness

The robustness of the method was evaluated by making systematic adjustments to various experimental conditions, including the column oven temperature, mobile phase composition, RT, theoretical plates (TPs), and peak area. The mobile phase consisted of 55% methanol (*X*) and 45% TFA (*Y*). The column oven temperature was maintained at 30°C, with a variation of ±2°C. The effect of these changes on recovery percentages, RTs, and repeatability was assessed to evaluate method robustness [26–28]:

(3) TP N=16tRW2.

### 2.11. Specificity by Forced Degradation

Specificity was assessed by subjecting samples to forced degradation under various conditions, including base hydrolysis, acid hydrolysis, oxidation, warm air degradation, and photodissociation (using dry heat, UV treatment, and peroxide solution). These forced degradation studies help identify potential interferences and validate the stability of the assay. A DAD‐equipped HPLC system was used for analysis. Forced degradation was employed to establish the assay’s ability to indicate the stability of dasatinib under various stress conditions [29, 30].

### 2.12. Analysis of Dasatinib From SMEDDS

#### 2.12.1. Preparation of SMEDDS

**FIGURE 2:**
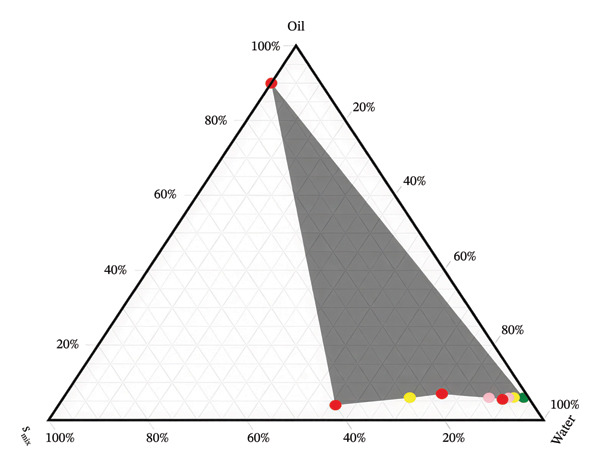
Ternary phase diagram for the preparation of SMEDDS of dasatinib.

Dasatinib’s solubility was used to select the appropriate surfactant (Tween 40), co‐surfactant (Transcutol HP), and oil (Capryol 90) for the SMEDDS formulation. A ternary phase diagram (Figure 2) was used to determine the optimal ratios of these components. In Formulation I, 6 g of surfactant and co‐surfactant, 4 g of oil, and 50 mg of dasatinib were combined. Formulation II contained 7 g of surfactant and co‐surfactant, 3 g of oil, and 50 mg of dasatinib. The formulations were stored in tightly sealed glass tubes at room temperature (25°C) until required [19].

#### 2.12.2. Extraction of Dasatinib From SMEDDS

The methodology includes measuring the dasatinib concentration in several SMEDDS formulations. By combining 5 mL of the formulation with 20 mL of methanol, the drug concentration of the dasatinib‐loaded SMEDDS formulation was ascertained. The sample was homogenized, dissolved, and centrifuged for 15 min at 25°C at 3000 rotations per minute. The 0.2 mL supernatant was filtered through a 0.45‐µm membrane filter and combined with the methanol before HPLC analysis [19].

## 3. Results

### 3.1. Method Development

The method development for quantifying dasatinib started with optimizing the mobile phase. The mobile phase consisted of two components: *X*, which included methanol, and *Y*, which contained 0.1% TFA. A range of chromatographic conditions was tested, including variations in mobile phase composition, column temperature, and flow rate, to achieve well‐defined peaks and satisfactory RTs. Several proportions of the mobile phase components (*X* and *Y*), such as 50:50 v/v, 55:45 v/v, and 60:40 v/v (as shown in Table 2), were evaluated based on key chromatographic parameters, including RT, tailing factor, TPs, and peak purity.

**TABLE 2:** Method development—mobile phase optimization.

Parameters	Acceptance value	Results		
Mobile phase *X* (%): mobile phase *Y* (%)	—	50:50	55:45	60:40
Dasatinib retention time (min)	—	6.91	4.95	3.97
Tailing factor	0.8–1.5	1.19	1.15	1.17
Theoretical plates (*N*)	> 2000	6315	6647	7116
Peak purity	1	1	1	1

During the screening process, the initial mobile phase proportion of 10:90 v/v resulted in dasatinib being eluted after 10 min. By gradually increasing the methanol (*X*) concentration from 20%–60% and simultaneously decreasing TFA (*Y*) from 80%–40%, the RT decreased, and sharper peaks were observed with no tailing. The optimal mobile phase composition was found to be 55:45 v/v, providing the best results in terms of peak symmetry and RT. A flow rate of 1 mL/min was selected for the analysis of dasatinib, yielding improved chromatographic performance. A change in flow rate does not change peak purity. A flow rate of 1 mL/min was selected for the analysis of dasatinib.

### 3.2. Linearity and Sensitivity Studies

**FIGURE 3:**
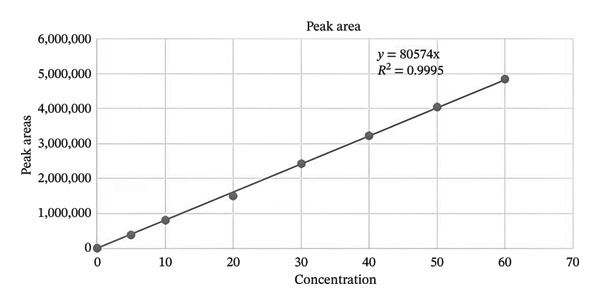
Calibration curve of dasatinib.

The resulting calibration curves revealed linearity in the dose range of 10–60 µg/mL (Figure 3). Dasatinib’s highest regions were plotted against the corresponding amount, and the resulting curves were then subjected to a linear regression measurement. A linear relationship was found (Figure 2) in the concentration range from 10–60 µg/mL. The linear equation *Y* = 80,578 + C obtained from experiments and correlation coefficients (goodness‐of‐fit) of 0.9998 were found in the dose ranges under investigation. Analysis of the resulting graph yielded a value of 0.9995 for the coefficient of determination or *R* ^2^. So, a good correlation was observed between the concentration of dasatinib and the peak area.

The LOQ and LOD were used in sensitivity experiments. LOD and LOQ values from the experiment were 0.17 and 0.50 µg/mL, respectively. To evaluate the accuracy and dependability of the procedure at the LOQ, three samples were prepared and subsequently subjected to three days of analysis. A mean value of 2.03 ± 0.02 µg/mL was obtained from the average results, and the intra‐ and interday precision displayed coefficients of variation (CVs) < 2%. Response factor for 10 µg/mL was 80,826.5 units per ppm, 20 µg/mL was 75,351.8 units per ppm, 30 µg/mL was 80,764.5 units per ppm, 40 µg/mL was 80,798 units per ppm, 50 µg/mL was 80,817.1 units per ppm, and 60 µg/mL was 80,840.8 units per ppm. Results of response factor show good linearity and sensitivity of the analytical method across the tested range.

### 3.3. Identification Studies

Furthermore, the data from the identification studies indicated the presence of dasatinib. The observation suggested detecting the investigation compound (dasatinib) in intermediate and method precision at 4.97 min of RT.

### 3.4. Precision Studies

The values for intraday and interday precision are shown in Table 3. The same set of six samples was tested to assess the precision. By selecting one of the quantities (50 µg/mL) from the linearity curve, instrument precision was attained (Table 4). It was observed that the asymmetry, RT, and TP values for the experimental medication were consistent.

**TABLE 3:** Intraday and interday precision data of dasatinib.

Sr. no.	Concentration	Intraday precision area	Interday precision area		
			Day 1	Day 2	Day 3
1	50 µg/mL	4021891	4021895	4021887	4021894
2		4068713	4068713	4068711	4068716
3		4021186	4021183	4021187	4021181
4		4023256	4023250	4023259	4023255
5		4039746	4039740	4039747	4039741
6		4055978	4055974	4055980	4055972
Mean		4038462	4,038,459	4038461	4038459
Standard deviation		20138.83098	20138.821489	20138.624625	20139.140313
RSD		0.50	0.499	0.499	0.499

**TABLE 4:** Instrument precision data of dasatinib.

Sr. no	Area	Retention time	Theoretical plates	Asymmetry	% assay
1	4043855	4.97	6586	1.19	99.60
2	4058746	4.97	6547	1.15	99.97
3	4078774	4.97	6522	1.20	100.46
4	4057441	4.97	6351	1.18	99.93
5	4049116	4.97	6322	1.15	99.73
6	4065742	4.97	6497	1.16	100.14

### 3.5. Accuracy Studies

A freshly created calibration curve was used to interpolate duplication peak regions taken from five similar standard solutions to assess accuracy. Table 5 presents the RSD values, which were established to be 0.27, 0.73, and 0.45 at 80%, 100%, and 120% spiked levels, respectively.

**TABLE 5:** Accuracy data of dasatinib.

% level spiked	Reps	Spiked concentration (µg/mL)	Area	Amount recovered (µg/mL)	% recovery	RSD
80	1	40.00	3231922	39.80	99.51	0.27
	2	40.00	3214479	39.59	98.97	
	3	40.00	3224586	39.71	99.28	

100	1	50.00	4040855	49.76	99.53	0.73
	2	50.00	4058746	49.99	99.97	
	3	50.00	4098774	50.48	100.96	

120	1	60.00	4850452	59.74	99.56	0.45
	2	60.00	4864773	59.91	99.85	
	3	60.00	4821763	59.38	98.97	

### 3.6. Studied to Determine the Method’s Robustness

The observations from the dasatinib robustness trials are summarized in Table 6. Three different mobile phase compositions were tested to assess the robustness of the method. The TP values ranged from 5512–6754, indicating consistent column efficiency across different conditions. The RT remained nearly stable, with a value consistently around 4.97 min. The asymmetry of the peaks, which indicates peak shape, was measured and found to range between 1.10 and 1.29, reflecting minimal variation.

**TABLE 6:** Determination of robustness of dasatinib.

Condition	Area	Retention time	Theoretical plates	Asymmetry
*Mobile phase composition*				
Methanol 57—TFA 43	4022167	4.83	5512	1.21
Methanol 55—TFA 45	4040855	4.97	6586	1.19
Methanol 53—TFA 47	4042687	4.99	6754	1.25

*Column oven temperature*				
35°C temperature	4031789	4.97	6639	1.29
30°C temperature	4040855	4.97	6586	1.19
25°C temperature	4012117	4.97	6289	1.10

*Different laboratory*				
Methanol 55—TFA 45 (in quality assurance laboratory)	4040855	4.97	6586	1.19
Methanol 55—TFA 45 (in Pharmaceutics laboratory)	4040701	4.97	6571	1.19

To evaluate the reproducibility of the method across different laboratories, two separate quality assurance facilities were involved: the Agilent laboratory and the Pharmaceutics laboratory (Shimadzu). In both laboratories, the RT and asymmetrical values were consistent, demonstrating that the method produced similar results despite variations in instrumentation and laboratory studies. This confirms the robustness and reproducibility of the developed method under different experimental conditions.

### 3.7. Stability Studies

Dasatinib stability was assessed under five different stress conditions: acid, base, peroxide, UV exposure, and dry heat. The results are summarized in Table 7. The highest assay percentage of 92.53% was observed in the basic environment, with a corresponding degradation percentage of 7.47%. In contrast, UV exposure led to the lowest assay value of 85.65%, with a degradation percentage of 14.35%. Degradation was also observed under acidic, peroxide, and dry heat conditions, with degradation percentages of 11.71%, 11.58%, and 14.09%, respectively. These results indicate that dasatinib is more stable under basic conditions and more susceptible to degradation under UV and dry heat conditions.

**TABLE 7:** Stability studies of dasatinib.

Sample Id	Degradation condition	Area	% assay	Peak purity	% degradation
Control	NA	4059946	100.00	1.000	NA
Acid	1 M HCL, 1.0 mL, @ room temperature for 60 min	3584413	88.29	0.998	11.71
Base	1 M NaOH, 1.0 mL, @ room temperature for 30 min	3756474	92.53	0.996	7.47
Peroxide	30% H_2_O_2_, 1.0 mL, @ room temperature for 60 min	3487921	88.42	0.992	11.58
UV	254 nm for 16 h	3589755	85.65	0.990	14.35
Dry heat	110°C for 8 h in an oven	3477441	85.91	1.000	14.09

Dasatinib’s stability under acid degradation was evaluated using HPLC chromatograms, which revealed the presence of the molecule with a RT of 4.97 min. Similarly, base hydrolysis and dry heat degradation techniques also showed dasatinib at the same RT of 4.97 min. The molecule was consistently detected at 4.97 min in HPLC chromatograms when subjected to oxidation and photolytic degradation, further assessing the stability of dasatinib (Supporting Figures (available here)). Additionally, blank, placebo, dasatinib API, and microemulsion formulation samples were injected, revealing unknown degradation peaks. These additional observations provide further insights into the degradation profile of dasatinib under various stress conditions, enhancing the understanding of its stability.

### 3.8. Analysis of Dasatinib From SMEDDS

The assay findings of dasatinib‐embedded SMEDDs are shown in Table 8. The percentage test value for both formulations (I and II) was greater than 99.5%. Additionally, 0.021 was noted as the percentage RSD value for both formulations.

**TABLE 8:** Assay of dasatinib extracted from SMEDDS.

Sr. no.	Formulation	% assay	Mean (%)	RSD %
1.	I	99.64	99.66	0.021
		99.67		
		99.68		

2.	II	99.71	99.71	0.021
		99.70		
		99.74		

**FIGURE 4:**
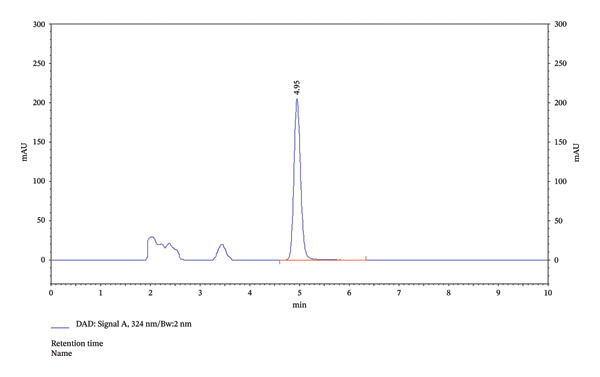
Dasatinib in lipid excipients.

In addition, the HPLC chromatogram was used to identify the presence of excipients that were detected along with the investigation drug. Dasatinib in the chromatogram was identified at a RT of 4.97 min (Figure 4).

## 4. Discussion

This study aimed to validate a novel HPLC method for detecting dasatinib in SMEDDSs. SMEDDS formulation was used to validate the analytical method. The results showed that dasatinib could be detected linearly in SMEDDS, with all samples demonstrating over 98% recovery and robustness. Additionally, dasatinib showed stability under various degradation conditions, including acidic, basic, UV light, and dry heat. The RSD values indicated a high degree of reproducibility, validating the novel HPLC method for detecting dasatinib in SMEDDS (Tables 2, 3, 4, 5, 6, 7, and 8, Figures 2, 3, and 5, Supporting figure).

FIGURE 5Identification graphs for the precision of the method. (a) Showing 100% level of 50 µg dasatinib (instrument precision); (b) intraday precision showing 4.97 of RT; (c) interday precision showing 4.97 of RT.

**Figure:**
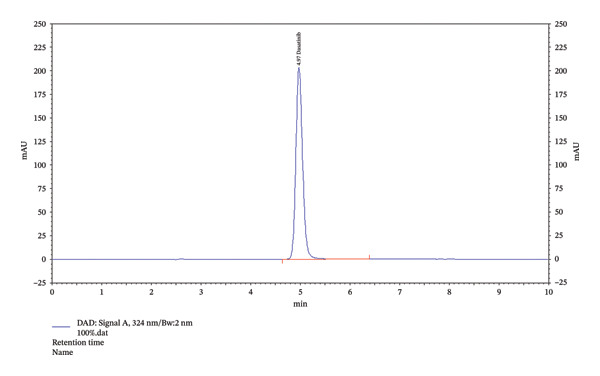
(a)

**Figure:**
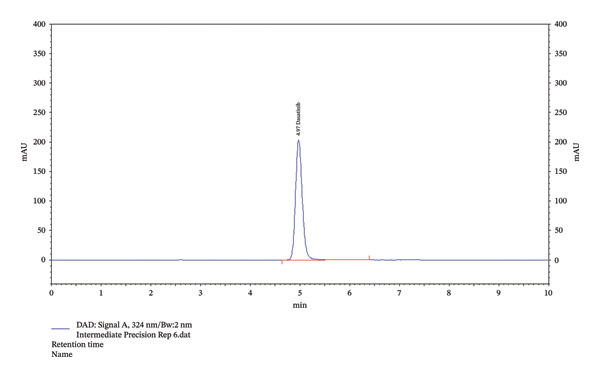
(b)

**Figure:**
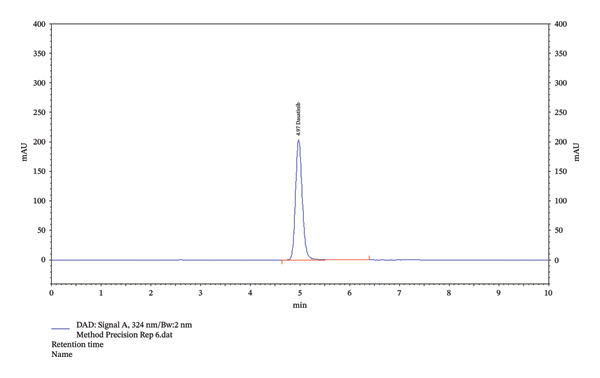
(c)

In this study, a validated HPLC method with DAD was employed to quantify dasatinib in various SMEDDS formulations. HPLC is a widely accepted analytical technique that enables precise separation, identification, and quantification of compounds in complex liquid matrices. It is routinely applied in pharmaceutical analysis, including the evaluation of investigational drugs like dasatinib in formulation systems [5, 12–14].

The model HPLC instrument used in this research was an Agilent Zorbax SB‐Aq C18 column, which is known for its enhanced efficiency and peak shape [15]. The results showed that the best chromatographic performance was achieved when the methanol content was increased to 55%, while the TFA concentration was reduced to 45%. The analyte was detected at 324 nm using UV detection. This specific mobile phase composition produced well‐resolved peaks with minimal tailing and an optimal RT. Dasatinib was found to have an RT of approximately 4.95 min, consistent with previous research that employed HPLC using methanol to efficiently detect the analyte [10]. Research has also indicated that the obtained peaks were well‐resolved, demonstrating the method’s adequate specificity [16].

Compared to previously reported methods (Table 1), the developed method exhibited several analytical and operational advantages, including shorter analysis time, a buffer‐free mobile phase composition, and high selectivity in lipidic matrices. These advantages make the method highly suitable for formulation laboratories where rapid throughput and matrix compatibility are critical. In addition, the ability to achieve accurate quantification without interference from surfactants or excipients establishes this work as a formulation‐specific analytical advancement rather than a conventional method validation study.

For precision assessment, dasatinib solutions at six different concentrations were analyzed in triplicate to evaluate intraday variability. The LOD and LOQ were calculated using the standard deviation from the calibration curve data [25]. Low CV values confirmed the method’s accuracy and consistency [26], aligning with previous studies validating its suitability for quantitative analysis [21, 22].

The International Council for Harmonization (ICH) guidelines on method robustness were followed, which state that robustness is achieved when chromatographic conditions, such as mobile phase composition changes (±2%) and column oven temperature adjustments (±2°C), remain within acceptable bounds to ensure suitable chromatographic results [27, 28]. The findings of this study aligned with previous research [24], showing that dasatinib’s RT and peak symmetry remained consistent over time and across different experimental conditions. The RP‐HPLC method was found to be both robust and reproducible for quantifying dasatinib [21].

In the stressed stability studies, dasatinib was isolated at appropriate intervals and subjected to HPLC analysis until a significant degree of degradation was observed. This approach aimed to detect any potential alterations in the sample’s appearance, such as color changes or the presence of precipitation [29]. Dasatinib demonstrated significant stability under harsh conditions, retaining its colorless appearance after 8 h of exposure to dry heat at 110°C, with only slight signs of degradation. These findings are consistent with earlier studies, which highlighted dasatinib’s stability and the method’s reliability in detecting it [30].

Dasatinib was found to be more stable under basic degradation conditions, showing minimal signs of breakdown compared to acidic conditions or exposure to peroxide [29]. When exposed to photolytic conditions, dasatinib underwent further degradation in its solid state, with the sample changing color from bright yellow to pale yellow. No notable color change was observed during oxidation, in line with previous studies on the drug [30]. Chromatographic analysis showed no significant alterations in the test compound, reinforcing the conclusion that minimal chemical degradation occurred [31, 32]. No significant change in the degradation of API and formulation was noticed. Only a trace of degraded or nondegraded excipient peak is seen in the chromatogram.

Three separate assays were conducted to evaluate dasatinib‐containing SMEDDS preparations, all demonstrating excellent reproducibility (%RSD 0.021) and 100% recovery (Table 7). These results confirm that the validated HPLC method accurately quantifies dasatinib concentrations, even in the presence of excipients or within complex lipid‐based systems (Supporting Figures) [18, 33, 34]. The method is suitable for both resource‐limited laboratories and high‐throughput settings, making it applicable for routine quality control, large‐scale screening, and clinical investigations. Importantly, the proposed method expands analytical applicability by providing a reliable tool for quantifying dasatinib in lipidic nanoformulations—a domain not addressed by existing HPLC procedures. This establishes the method’s novelty and translational value in formulation research, bridging the gap between analytical chemistry and drug delivery sciences. Furthermore, the method’s simplicity, speed, and robustness make it ideal for formulation optimization and routine stability testing, thereby contributing to pharmaceutical development practices where such validated methods are lacking. However, further studies are required to evaluate the formulation’s stability against enzymatic degradation and to confirm its efficacy under in vivo conditions.

## 5. Conclusion

A cost‐effective and validated HPLC‐UV method was developed for the quantification of dasatinib, demonstrating excellent resolution, a short runtime (∼5 min), and robust validation parameters (linearity, precision, sensitivity, and accuracy). This method effectively separates dasatinib from SMEDDS components, addressing a previously unreported analytical need for such formulations. Its reliability supports routine and research applications in lipid‐based drug delivery systems. Moving forward, preclinical and clinical studies should evaluate the safety, efficacy, and pharmacokinetics of dasatinib‐loaded SMEDDS, while formulation optimization and regulatory validation could further bridge the gap between analytical innovation and therapeutic impact in CML treatment.

## Funding

The authors would like to acknowledge the funding from the Ongoing Research Funding Program (ORF‐2026‐146), King Saud University, Riyadh, Saudi Arabia.

## Conflicts of Interest

The authors declare no conflicts of interest.

## Supporting Information

Figure S1: Figure S1: Degradation of Blank, Placebo, Formulation, and Dasatinib API by HCL.

Figure S2: Degradation of Blank, Placebo, Formulation, and Dasatinib API by NaOH.

Figure S3: Degradation of Blank, Placebo, Formulation, and Dasatinib API by Dry heat.

Figure S4: Degradation of Blank, Placebo, Formulation, and Dasatinib API by Oxidation.

Figure S5: Degradation of Blank, Placebo, Formulation, and Dasatinib API by UV.

## Supporting information


**Supporting Information** Additional supporting information can be found online in the Supporting Information section.

## Data Availability

Data are included in the article/supporting information referenced in the article.
