# Supplementary material for: Development and Validation of a Robust RP‐HPLC Method for Quantifying Dasatinib in Self‐Microemulsifying Drug Delivery Systems
Source: Int J Anal Chem. 2026 Feb 1;2026:4376722. doi: 10.1155/ianc/4376722 (PMC12862106; doi:10.1155/ianc/4376722)
Supplement: Supplementary file 1 — Supporting Information Additional supporting information can be found online in the Supporting Information section. [file IANC-2026-4376722-s001.docx]

**Development and Validation of a Robust RP-HPLC Method for Quantifying Dasatinib in Self-Microemulsifying Drug Delivery Systems**

**Nitin. V. Kokare^*1^, Rohit. R. Shah^2^, Rana Salman Saad Al-Rashidi^3^, Kuntal Das^4^, Syed Mohammed Basheeruddin Asdaq^5^*,** **Walaa F. Alsanie^6^,** **Abdulhakeem S. Alamri^6^,** **Majid Alhomrani^6^,** **Amal F. Alshammary^7^, Syed Arif Hussain^8^, Syed Imam Rabbani^9^, Hanumantharayappa Bylappa^10^,** **Sultan Alshehri^11^**

^1^Department of Quality Assurance, Appasaheb Birnale College of Pharmacy, Sangli, 416416, Maharashtra, India, [nitinkokare111@gmail.com](mailto:nitinkokare111@gmail.com)

^2^Department of Pharmaceutics, Appasaheb Birnale College of Pharmacy, Sangli, 416416, Maharashtra, India, [rohitrshah@yahoo.co.in](mailto:rohitrshah@yahoo.co.in)

^3^Department of Pharmacy, King Khalid University Hospital, Riyadh, 11472, Saudi Arabia, [ranasalman2277@gmail.com](mailto:ranasalman2277@gmail.com)

^4^Research Director, Mallige College of Pharmacy, #71, Silvepura, Chikkabanavara Post, Bangalore, 560090, Karnataka, India, [drkkdsd@gmail.com](mailto:drkkdsd@gmail.com) (KD)

^*^^5^Department of Pharmacy Practice, College of Pharmacy, AlMaarefa University, Ad Diriyah 13713, Saudi Arabia, sasdaq@gmail.com (SMBA)

^6^Department of Clinical Laboratory Sciences, The faculty of Applied Medical Sciences, Taif University, Taif, 21944, Saudi Arabia, [w.alsanie@tu.edu.sa](mailto:w.alsanie@tu.edu.sa) (WFA); [a.alamri@tu.edu.sa](mailto:a.alamri@tu.edu.sa) (ASA); [m.alhomrani@tu.edu.sa](mailto:m.alhomrani@tu.edu.sa) (MA)

^7^Department of Clinical Laboratory Sciences, College of Applied Medical Sciences, King Saud University, Riyadh, 11472, Saudi Arabia, [aalshammary@ksu.edu.sa](mailto:aalshammary@ksu.edu.sa)

^8^Department of Respiratory Care, College of Applied Sciences, AlMaarefa University, Dariyah 13713, Riyadh, Saudi Arabia, [pulmoarif@gmail.com](mailto:pulmoarif@gmail.com)

^9^Department of Pharmacology and Toxicology, College of Pharmacy, Qassim University, 51452 Buraydah, Saudi Arabia, s.rabbani@qu.edu.sa

^10^Dept of pharmacology, #71, Mallige College of Pharmacy, silvepura, chikkabanavara post. Bangalore -560090, India, [findraya@gmail.com](mailto:findraya@gmail.com)

^11^Department of Pharmaceutics, College of Pharmacy, King Saud University, 11451 Riyadh, Saudi Arabia, [Salshehri1@ksu.edu.sa](mailto:Salshehri1@ksu.edu.sa) (SA)

*** Author for Correspondence:****Prof. Dr. Syed Mohammed Basheeruddin Asdaq,**

Department of Pharmacy Practice,

College of Pharmacy, AlMaarefa University,

Dariyah, 13713, Riyadh, Saudi Arabia, [sasdaq@gmail.com](mailto:sasdaq@gmail.com)


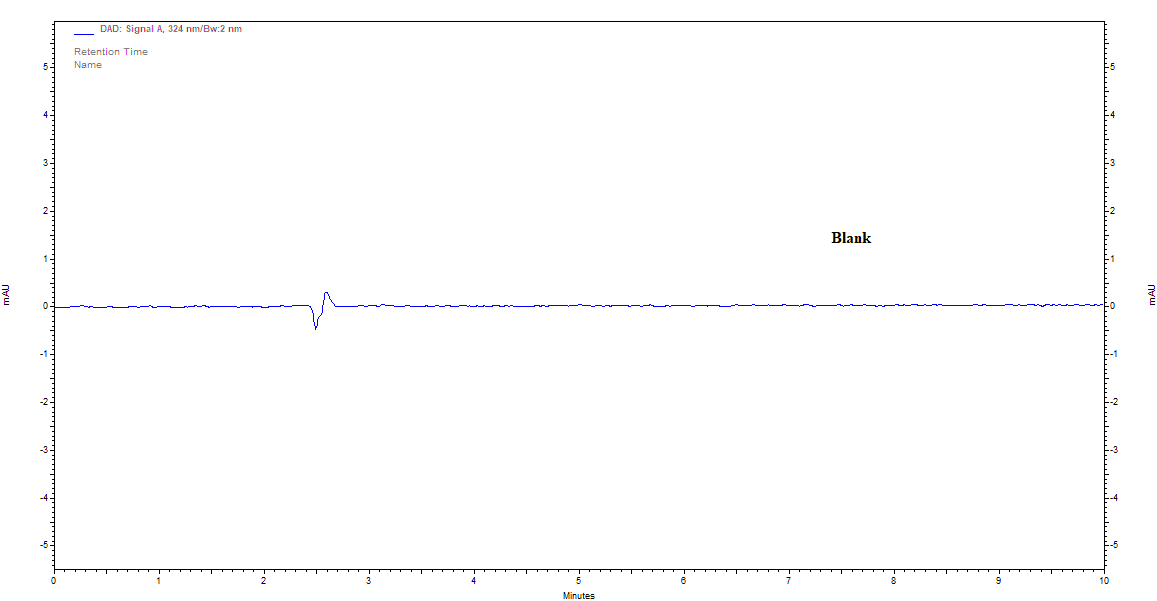


Blank for Acid hydrolysis


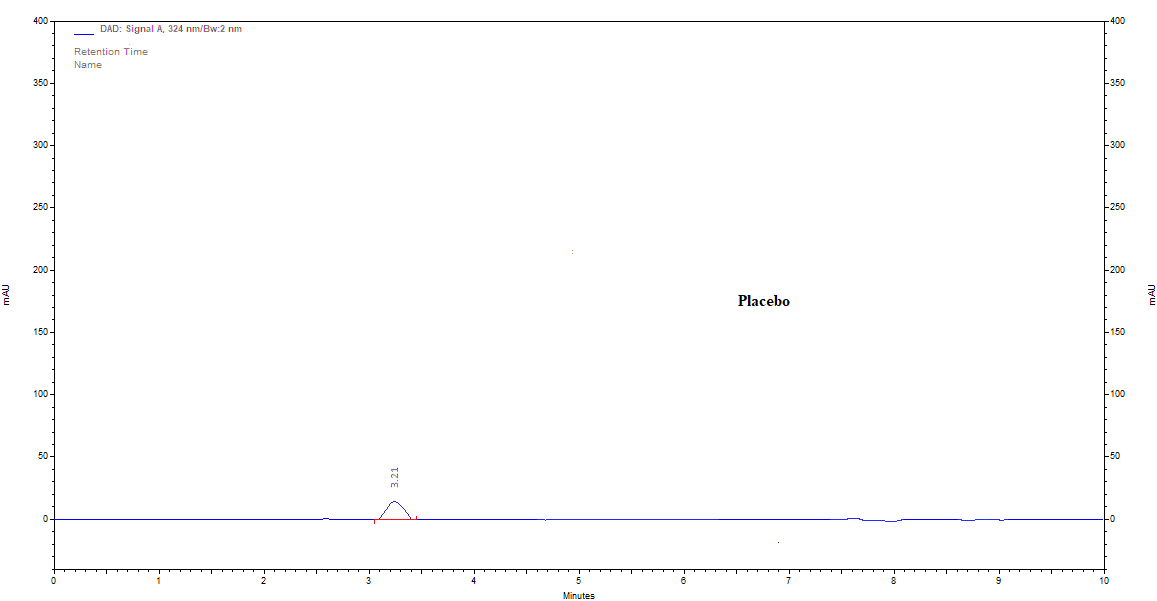


Placebo for Acid hydrolysis


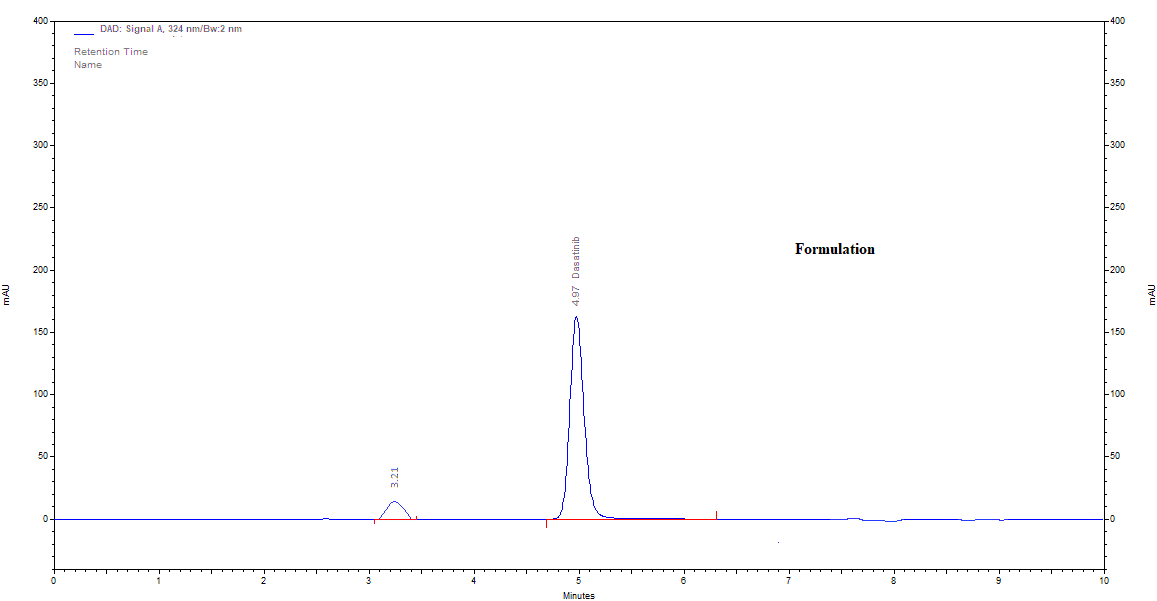


Microemulsion formulation for Acid hydrolysis


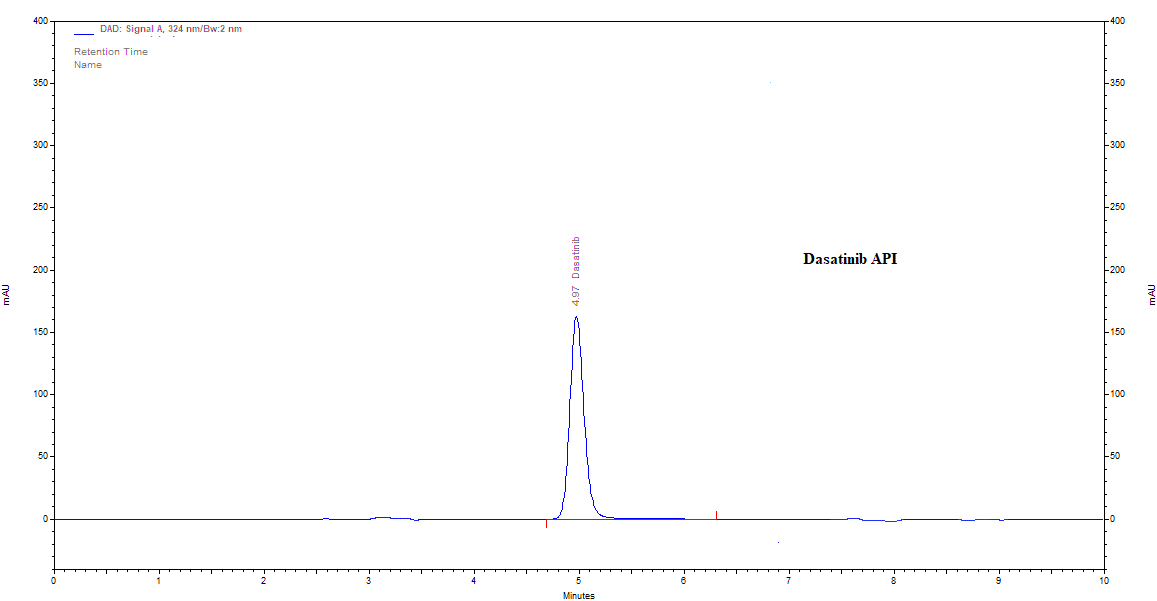


Dasatinib API for Acid hydrolysis

**Figure S1: Degradation of Blank, Placebo, Formulation, and Dasatinib API by HCL**


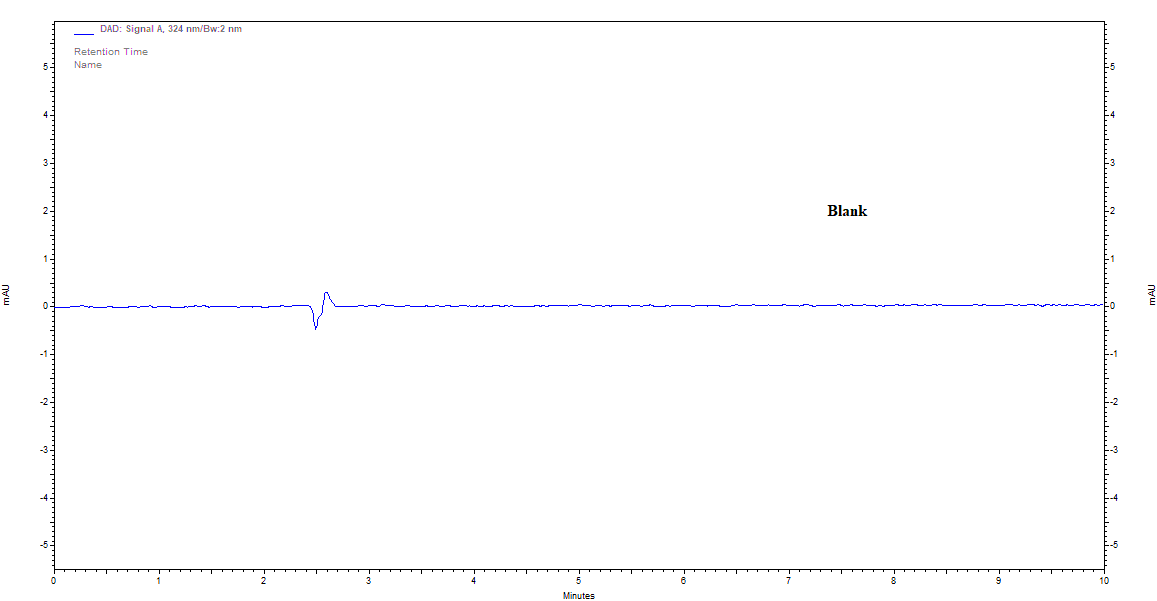


Blank for base hydrolysis


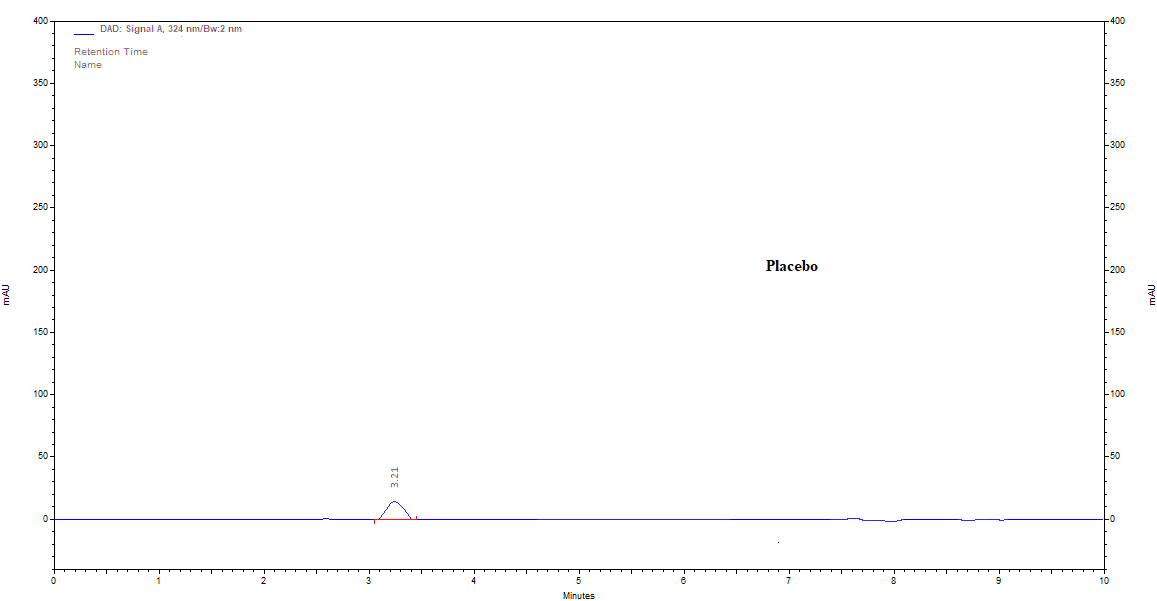


Placebo for base hydrolysis


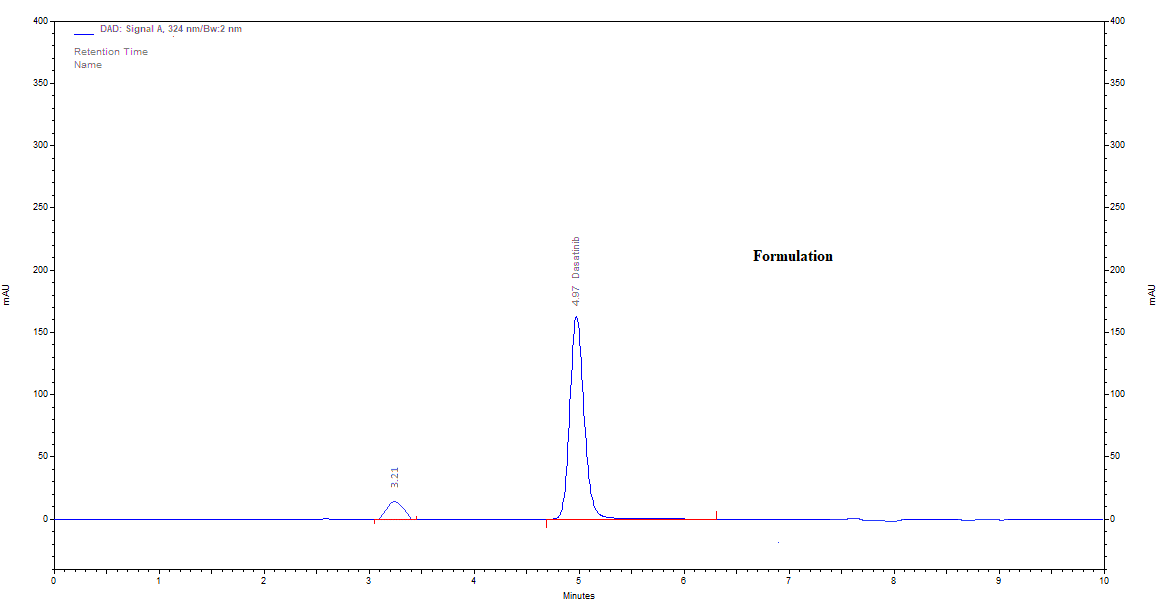


Microemulsion formulation for base hydrolysis


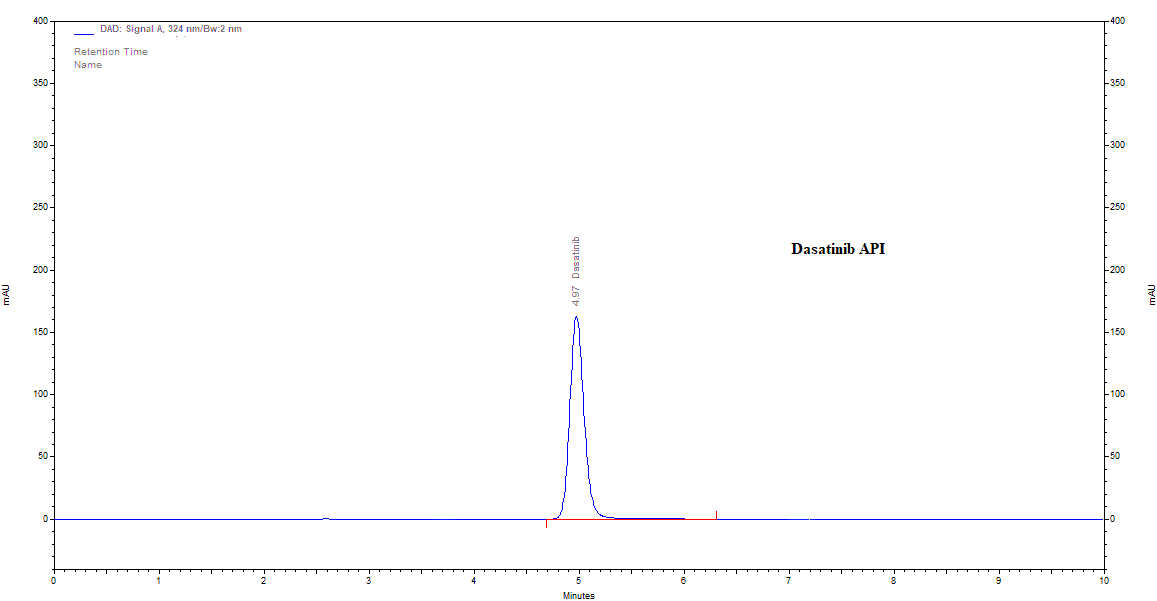


Dasatinib API for base hydrolysis

**Figure S2: Degradation of Blank, Placebo, Formulation and Dasatinib API by NaOH**


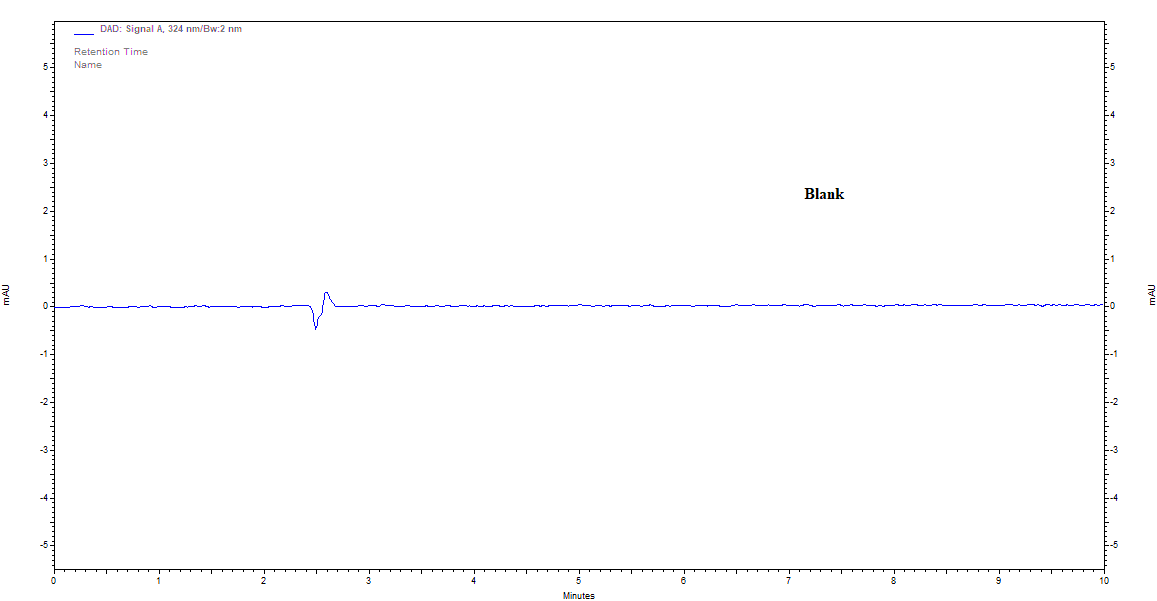


Blank for dry heat degradation


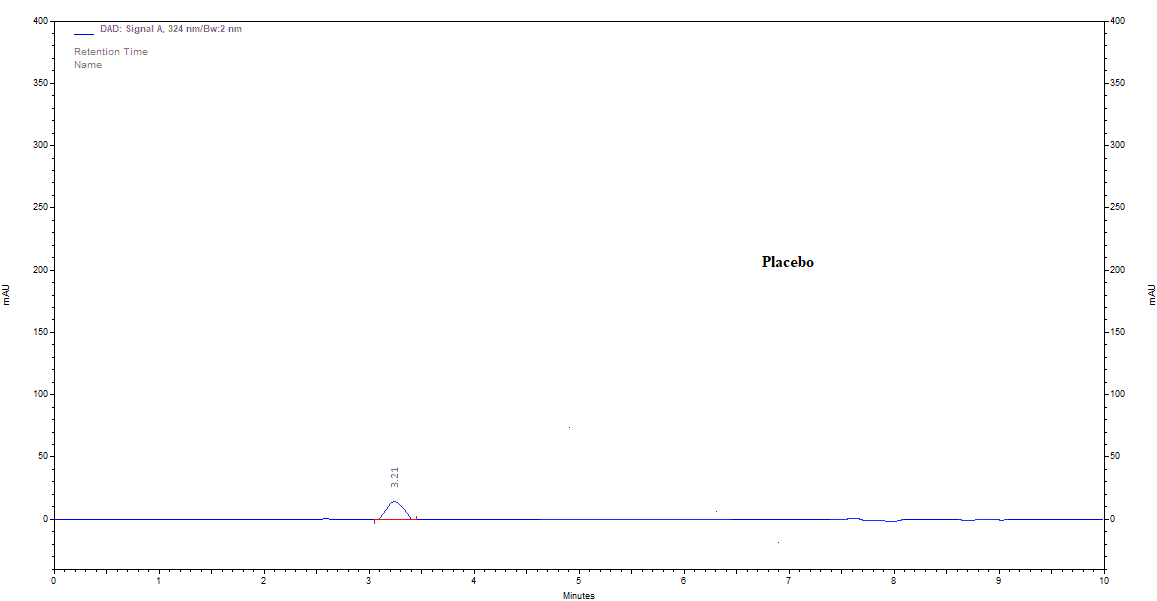


Placebo for dry heat degradation


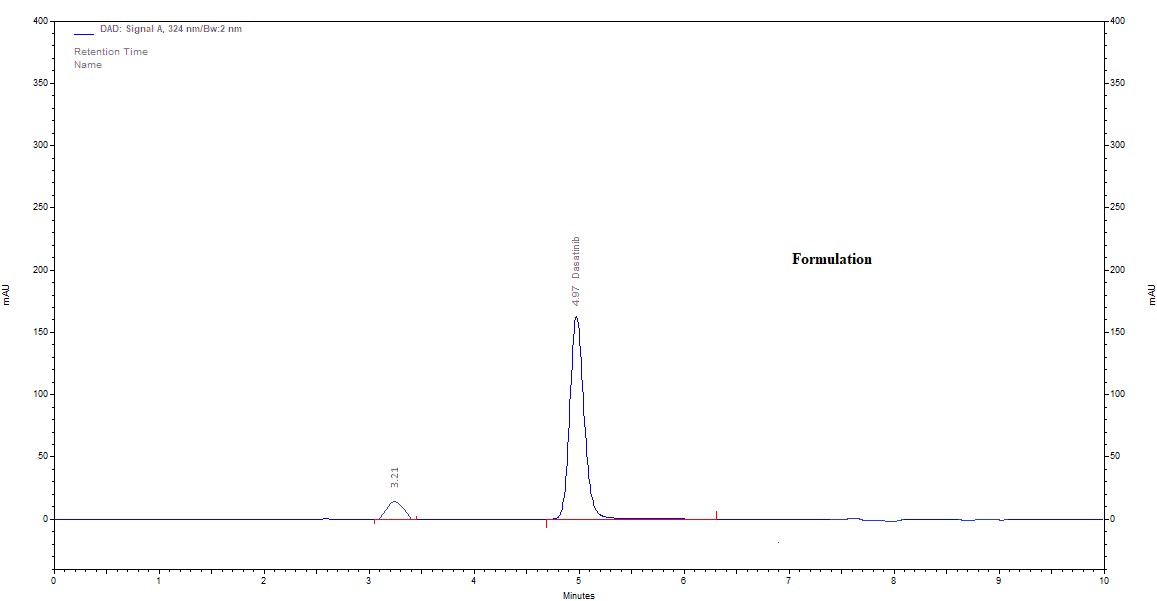


Microemulsion formulation for dry heat degradation


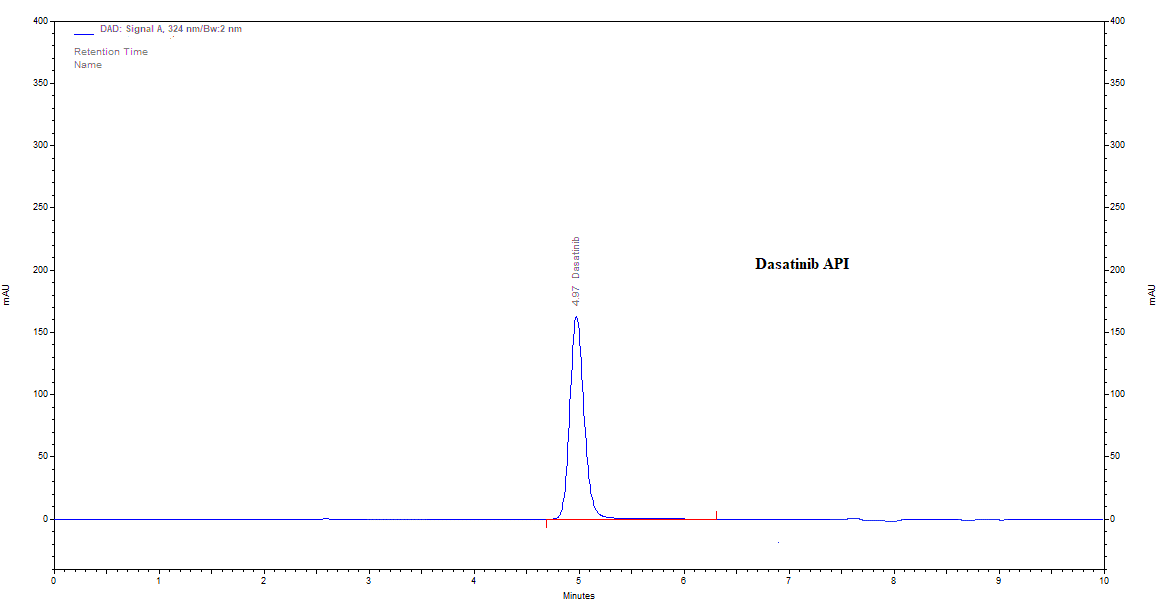


Dasatinib API for dry heat degradation

**Figure S3: Degradation of Blank, Placebo, Formulation and Dasatinib API by Dry heat**


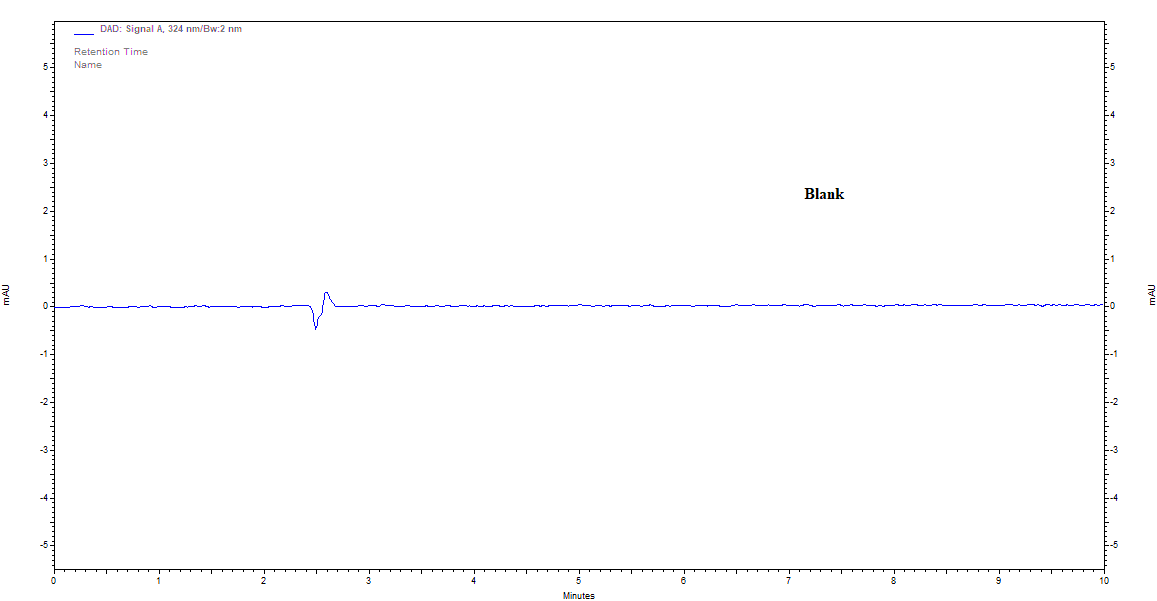


Blank for Oxidation degradation


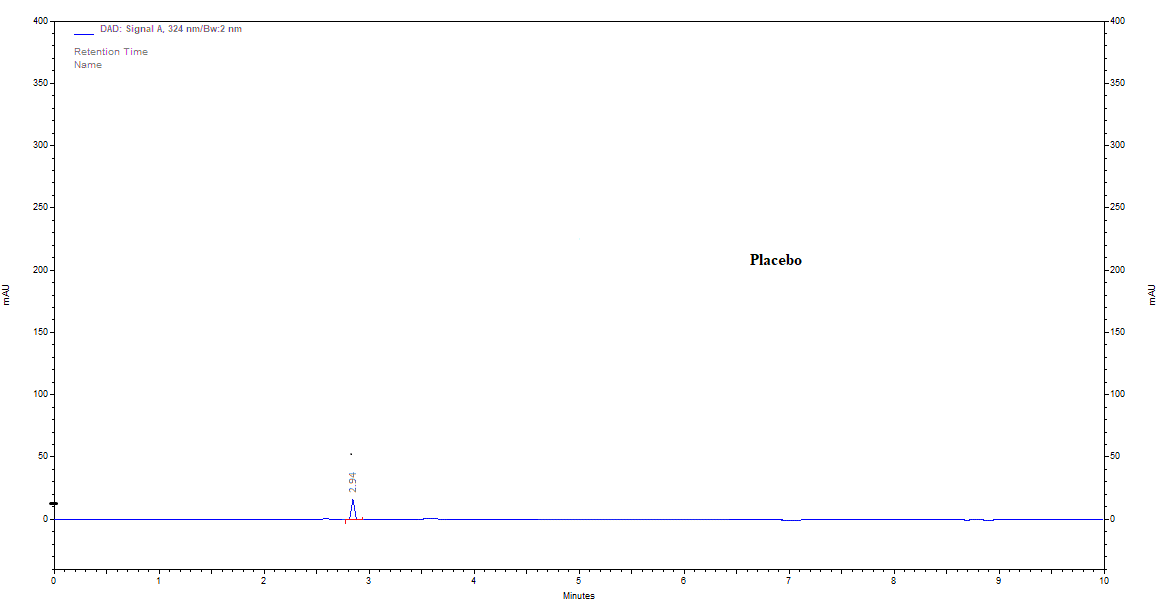


Placebo for Oxidation degradation


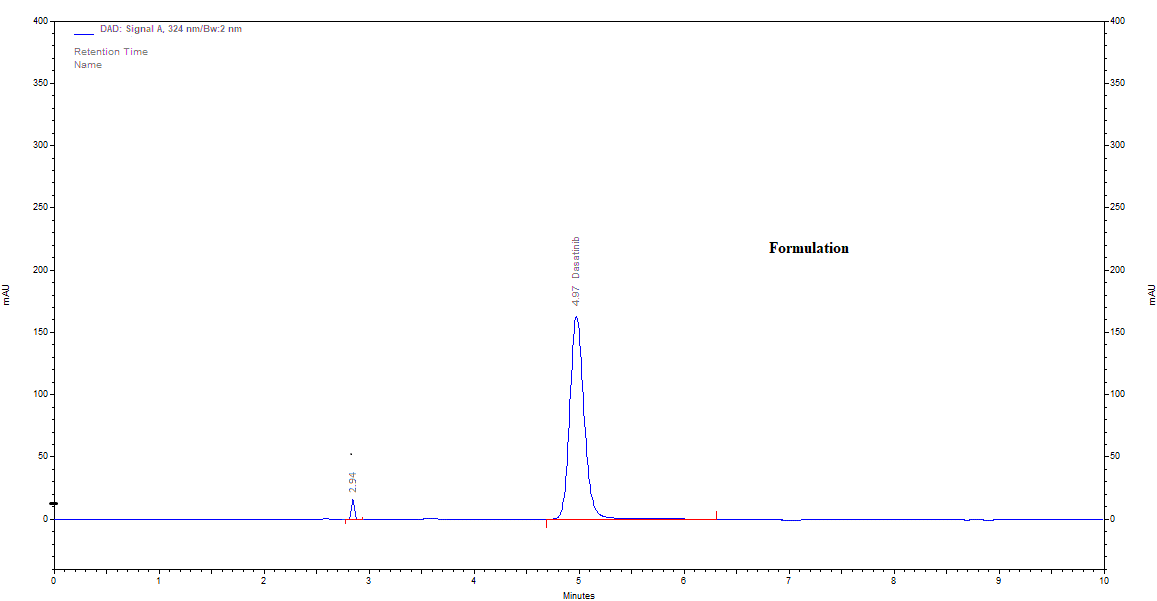


Microemulsion formulation for Oxidation degradation


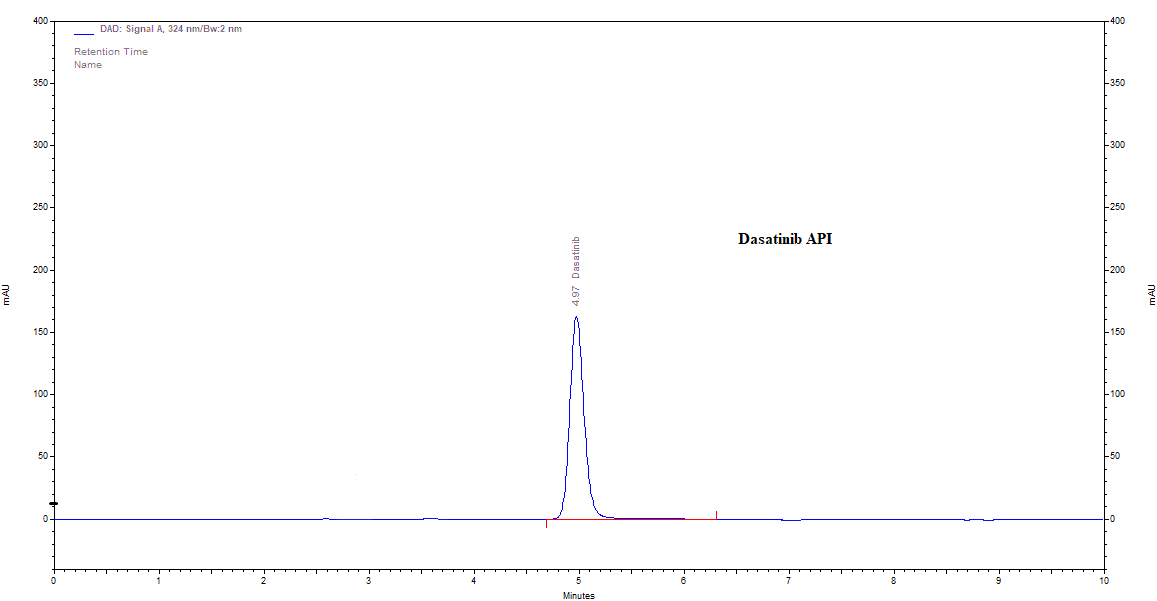


Dasatinib API for Oxidation degradation

**Figure S4: Degradation of Blank, Placebo, Formulation, and Dasatinib API by Oxidation**


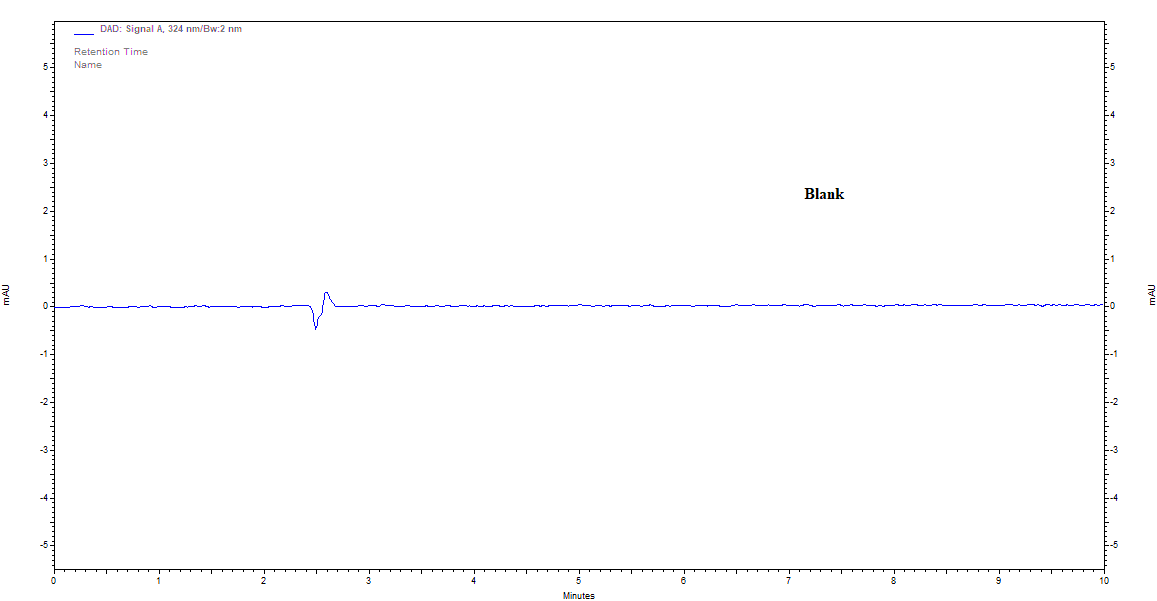


Blank for Photolytic degradation


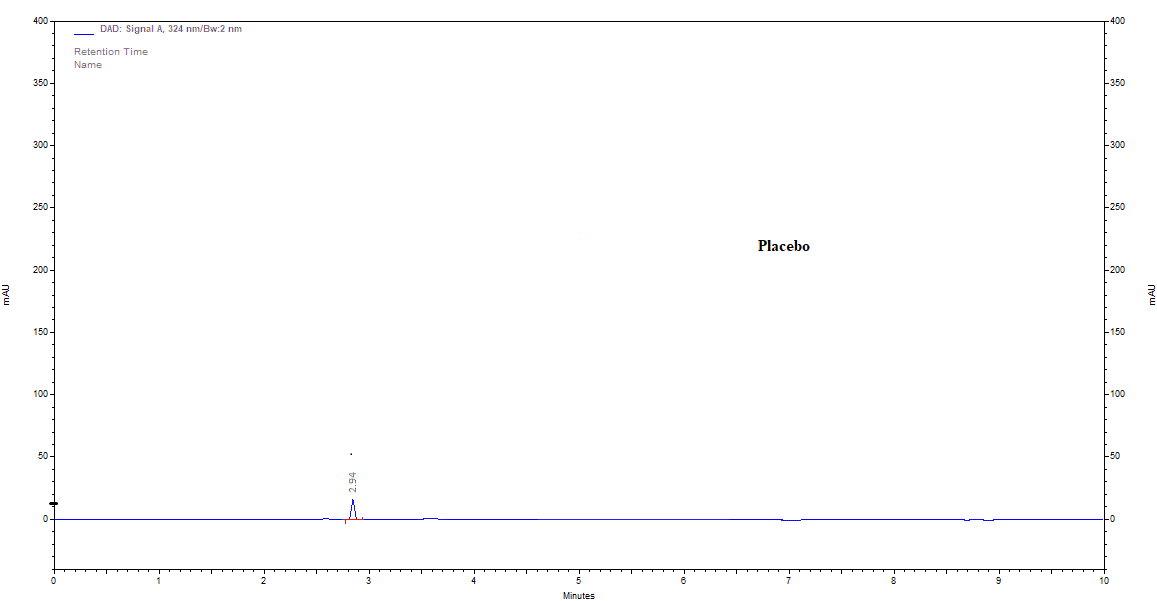


Placebo for Photolytic degradation


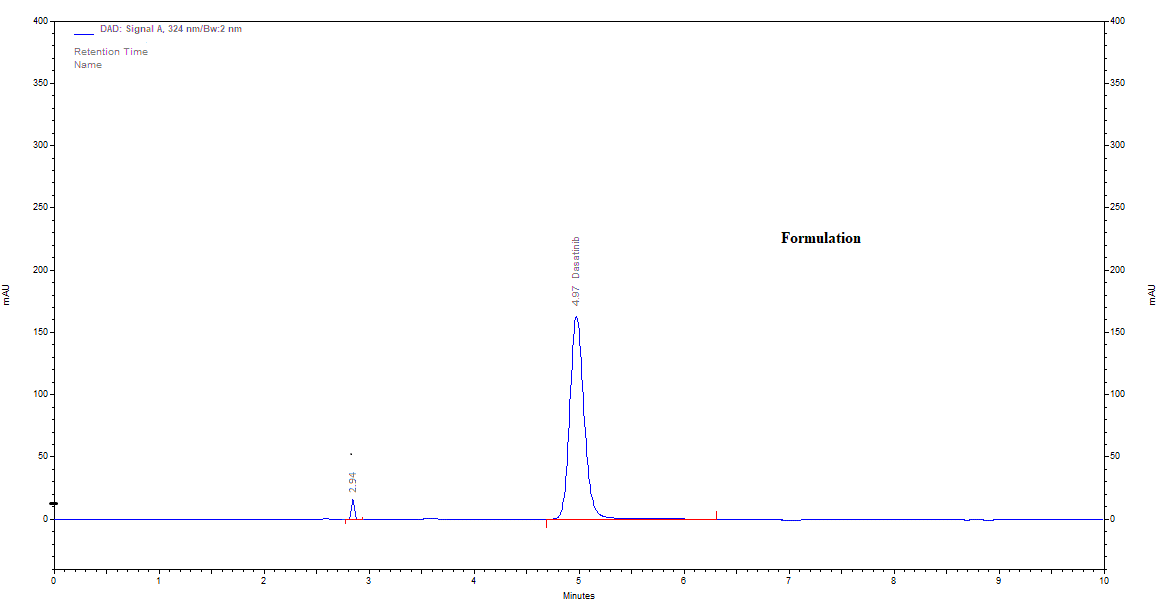


Microemulsion formulation for Photolytic degradation


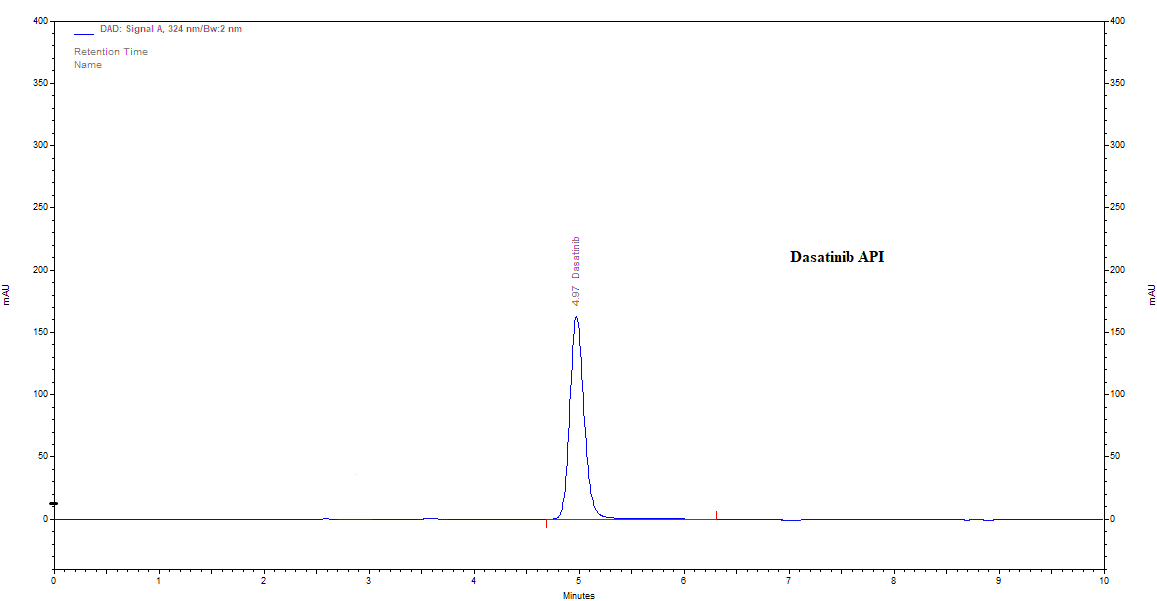


Dasatinib API for Photolytic degradation

**Figure S5: Degradation of Blank, Placebo, Formulation and Dasatinib API by UV**
